# Bicuspid Morphology and Rapid Deployment Valve Replacement: Is This Still a Contraindication?

**DOI:** 10.3390/jcm12237390

**Published:** 2023-11-29

**Authors:** Julia von der Linden, Florian Herrmann, Sergey Belyaev, Gerd Juchem, Sven Peterss, Christian Hagl, Alexey Dashkevich

**Affiliations:** 1 Department of Cardiac Surgery, Ludwig Maximilian University of Munich, 81377 München, Germanysven.peterss@med.uni-muenchen.de (S.P.);; 2 Leipzig Heart Center, Helios Clinic, University Clinic of Cardiac Surgery, 04289 Leipzig, Germany

**Keywords:** rapid deployment, SAVR, bicuspid aortic valve, Sivers classification

## Abstract

When using rapid deployment surgical aortic valve replacement (RD SAVR) in treating bicuspid aortic valve morphology (BAV), several challenges are faced due to annular asymmetry. The Sievers classification has been traditionally used for the description of BAV morphology. In this study, we aimed to conduct a retrospective feasibility analysis of RD SAVR in relation to the Sievers classification. From January 2014 to March 2022, 31 patients with BAV morphology (15 patients with Sievers type 0 BAV and 16 with Sievers type 1 BAV) underwent RD SAVR. Specific surgical techniques were applied depending on the BAV morphology. Comparable clinical outcomes were observed. No paravalvular leaks and no valvular re-interventions occurred in either group. CPB and cross-clamping times, as well as the prosthesis sizes used, were also not significantly different. Postoperative mean gradients were comparable in both groups. No significant distinction was found between the groups in terms of postoperative pacemaker indication, postoperative stroke, or death. Annular symmetry can be adequately restored through precise prosthesis sizing and placement according to an individual’s valve morphology regardless of the Sievers classification of BAV by choosing a different landmark for the initial suture. RD SAVR seems to be a safe approach for any bicuspid morphology, with good hemodynamic results and time-saving potential in experienced hands.

## 1. Introduction

**Figure 1:**
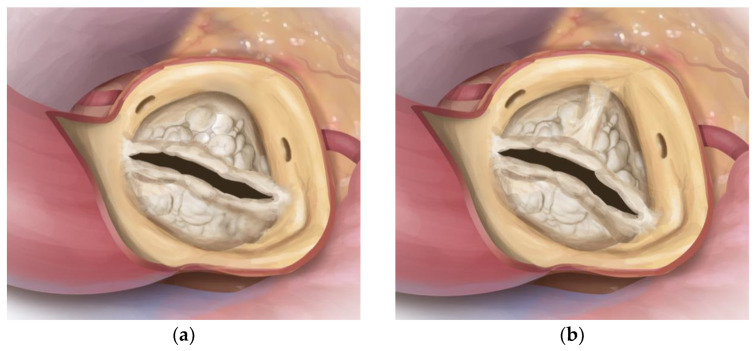
Drawing illustrating calcified BAV (BAV) of Sievers type 0 (**a**) and type 1, R-L (**b**). Source: Rob Flewell, 2023.

Surgical aortic valve replacement (SAVR) is the current therapeutic gold standard for aortic valve disease when indicated [1]. BAV morphology, the most common congenital valve abnormality, with an incidence of 1–2%, is the cause of valve stenosis in 50% of isolated aortic valve replacements and is associated with aortic dilatation, sinus asymmetry, commissural height differences, and altered geometry of the aortic annulus, as well as aortic valve endocarditis. Asymmetric morphology leads to fibro calcifications in most of the affected patients and, further, to aortic valve stenosis staring from the age of 50 years, much earlier than with tricuspid valve morphology. It requires earlier surgical intervention than tricuspid aortic valves and often concomitant aortic replacement [2,3,4]. In addition, patients with BAV have a higher risk of infective endocarditis and aortic root abscesses [5]. There are several different classifications or nomenclatures for bicuspid valves, including the Sievers and Schmidtke classification of 2007, by which the patient data in this paper were classified. There are three types of bicuspid morphology according to the number of raphes: in type 0, no raphes are present, and the two pockets are either antero-posterior (AP type) or arranged laterally. One raphe is found in type 1, either positioned between the right and left sinuses (R-L), the right sinus and the noncoronary sinus (R-N), or between the left and noncoronary sinuses (L-N) (Figure 1).

**Figure 2:**
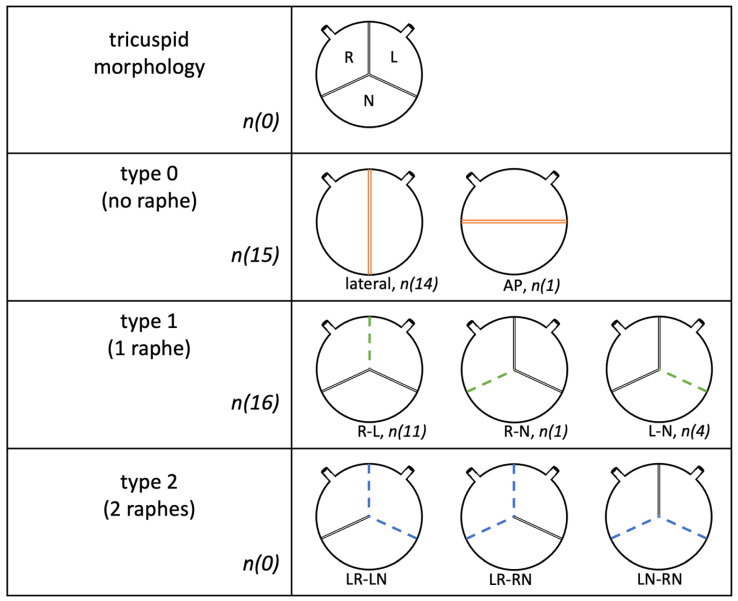
Sievers classification of BAV.

In type 2, there are two raphes: L-R or R-N [6] (Figure 2).

Previous studies have shown that prolonged aortic clamping time and cardiopulmonary bypass time are associated with increased postoperative morbidity and mortality [7,8]. RD prosthetic valves, which are almost suture-free and balloon-expanding forms of RD SAVR, were introduced as early as 2009 and offer the advantage of faster implantation, reduced aortic clamping and cardiopulmonary bypass time, and new possibilities in terms of reducing the size of the access pathway [9].

Currently, there are few data on the use of sutureless SAVR for treating bicuspid morphology and almost none on RD SAVR, which is why its use is controversial in the literature. Some studies with very small populations report postoperative results that are at least comparable to conventional SAVR in experienced hands, while others warn of paravalvular leaks and the risk of the dislocation of the prosthetic valve [10,11,12,13].

In the so-called modified Delphi expert panel of 2016, it was stated that RD SAVR can be used in bicuspid types with at least one raphe (types 1 and 2) but is contraindicated in BAV type 0 (level C recommendation, strength IIa) [7].

We analyzed the immediate and short-term outcomes of patients who underwent RD SAVR with different bicuspid morphologies to assess whether RD SAVR can be used in any Sievers grading or whether it leads to specific complications depending on the type.

## 2. Materials and Methods

### 2.1. Study Population

From January 2014 to March 2022, 35 patients with BAV morphology (25 patients with aortic valve stenosis, 1 patient with aortic regurgitation, and 9 patients with combined aortic valve pathology) underwent RD SAVR, all of whom received an Edwards Intuity Elite valve.

According to the Sievers system, 15 patients were classified as having a type 0 bicuspid valve, 16 as having type 1, and none as having type 2. The subtypes of four patients could not be determined retrospectively; therefore, they were excluded. Of the patients with Sievers type 0 BAV, one had ap morphology, while the rest were of the lateral type. The patients with Sievers type 1 were divided into 11 with type R-L, 4 with type L-N, and one with type R-N.

We devised our two study groups in accordance with the Sievers classification.

An additional comparison group of 79 patients with tricuspid morphology (TAV) from the same observation period was formed. The group consisted of a collective of patients with similar preoperative data and concomitant procedures performed.

Data were collected retrospectively, and the mean follow-up time was 15 months.

### 2.2. Statistical Analysis

Consent for surgery was obtained from all patients, and further data collection took place as part of the patients’ routine post-operative care. After collection from internal hospital patient management programs, the data were anonymized and processed in RedCap and subsequently analyzed using IBM SPSS (IBM Corp. Released 2017. IBM SPSS Statistics for Windows, Version 25.0. Armonk, NY, USA: IBM Corp.). In addition, the EuroSCORE II and, if applicable, the STS score were calculated.

After testing for normal distribution using the Shapiro–Wilk test, the *p*-values of categorical variables, presented as numbers and percentages of occurrence, were calculated using the chi-square test or Fisher’s exact test, and those of continuous variables, reported as means ± standard deviation, were calculated using the *t*-test for independent samples or Mann–Whitney test. When a *p*-value < 0.05 was attained, the null hypothesis was rejected, and a significant difference was assumed.

### 2.3. Surgical Approach

After many years of experience with RD SAVR in tricuspid morphology, the standard technique of RD SAVR for BAV morphology was established. The surgeries were performed using standard anesthetic and surgical techniques, with complete median sternotomy in most patients.

**Figure 3:**
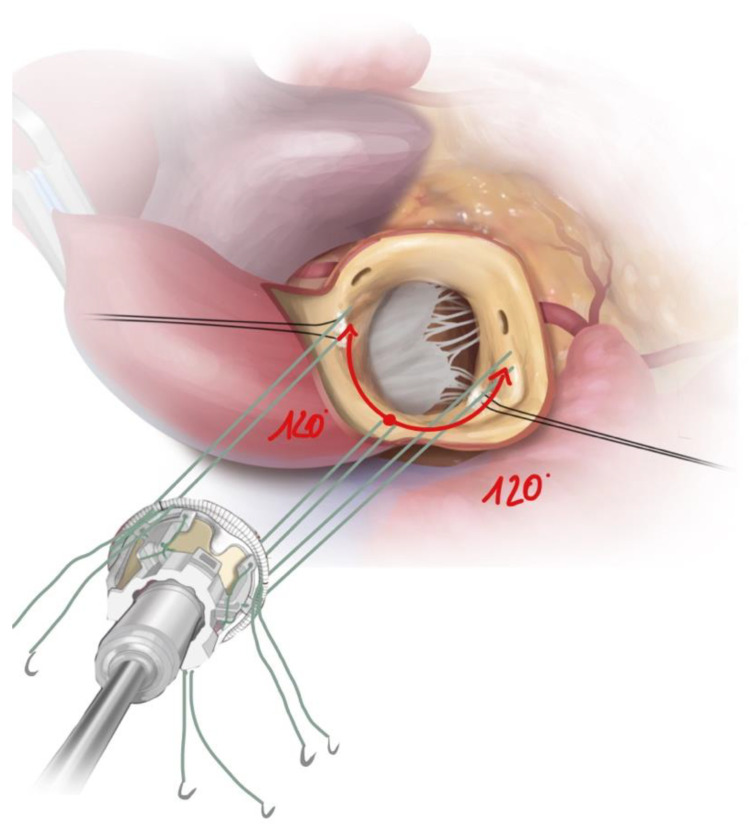
Drawing illustrating positioning of the guiding sutures in Sievers Type 1. BAV with leading suture in acoronary nadir, followed by two guiding sutures (red arrows) at a 120-degree offset. Source: Rob Flewell, 2023.

To reconstruct the geometry of the bicuspid annulus, the annular size was first measured using standard measuring devices (Edwards, CA, USA). Especially in the case of bicuspid morphology, the exact dimensioning of the annulus is crucial to avoid paravalvular leakage due to insufficient sealing of the usually elliptical annulus or to prevent higher-grade block patterns. The replica sizer was used to determine and mark two further suture points at a symmetrical distance of 120 degrees starting from a leading nadir. With Sievers Type 1, the leading nadir is usually located in the typically larger and prolaptic acoronary pocket (Figure 3).

**Figure 4:**
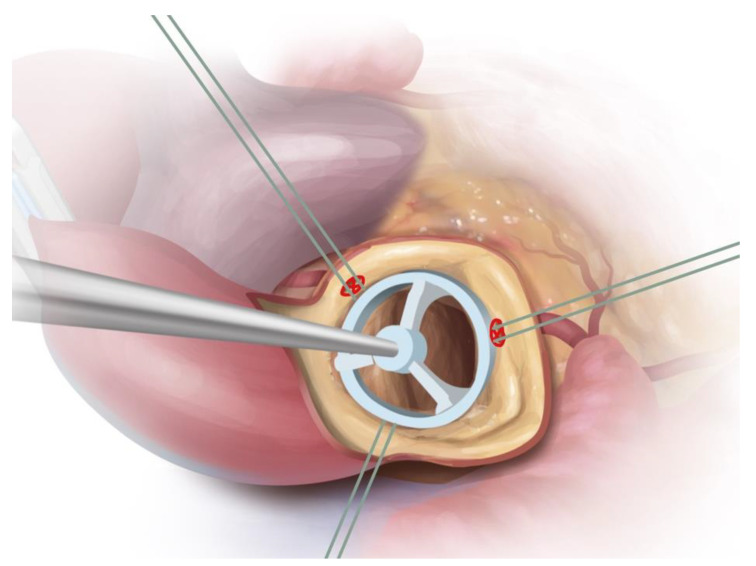
Drawing illustrating placement of guiding sutures in Sievers BAV type 0. Main guiding sutures are placed underneath coronary ostia to ensure a safe distance to prosthetic struts after implantation; the third guiding suture is placed at equal distance. Source: Rob Flewell, 2023.

In Sievers type 0, the leading suture point is placed at points in the free aortic annulus depending on the offset of the coronary arteries (they should not be covered by the prosthesis struts) and the remaining sutures at a symmetrical distance of 120 degrees with respect to the leading nadir. Here, special care must be taken to ensure the correct height of the landmarks so that the valve is not misaligned, creating a paravalvular gap. The nadirs are used to place three guiding sutures to implant the prosthetic valve alongside them into the root. The guiding sutures are tied after successful balloon expansion (Figure 4).

## 3. Results

### 3.1. Patient Characteristics

Patients in the Sievers type 0 group were on average 64.5 years old (SD 7.5 years) and thus not significantly younger than those with a type 1 morphology (62 years, SD 8.0 years). Interestingly, there were more women than men with type 1 BAV who received RD SAVR (56%), while only 13% with type 0 BAV were female.

Preoperative risk factors (e.g., smoking, diabetes, hypertension, obesity, hyperlipoproteinemia, and positive family history) were comparable in both groups, although those with BAV 0 presented, on average, with more risk factors per person (2.6 in BAV type 0, and 1.8 in BAV type 1).

To determine mortality risk, the New Euro Score II was calculated. There was no difference between the two groups, with averages of 2.9 ± 4 in BAV type 0 and 2.1 ± 1.2 in BAV type 1, the most common risk factors being arterial hypertension and hyperlipoproteinemia. Only one patient with type 1 BAV presented with a previous stroke (Table 1).

### 3.2. Valve Specific Data

Most patients in both cohorts presented with pure aortic valve stenosis; a combination with aortic valve regurgitation occurred in 33% of the BAV Type 0 patients and in 25% of those with BAV type 1. Only one patient (6%) of type 1 BAV and none with type 0 BAV showed pure insufficiency of the aortic valve. There was no significant difference in the preoperative gradients in both cohorts, with the mean maximum gradient for type 0 BAV being 67.0 ± 17.5 mmHg and that for type 1 BAV being 79.6 ± 24.3 mmHg. Both groups showed a significant reduction in aortic orifice area. Type 0 BAV resulted in a mean area of 0.85 ± 0.29 cm^2^; this value for type 1 BAV was 0.80 ± 0.26 cm^2^. Almost one third of the patients in both groups were also diagnosed with an aneurysm of the ascending aorta (27% in BAV type 0; 31% in BAV type 1). Among the BAV type 1 patients, only one had aortic valve surgery due to endocarditis, and one surgery was an emergency intervention (Table 2).

### 3.3. Intraoperative Data

Similarly, the majority in both groups had a complete sternotomy; 7% of patients with BAV type 0 and 19% with BAV type 1 underwent aortic valve replacement with a partial sternotomy. Most patients underwent concomitant procedures. In the BAV type 0 group, 33% underwent additional bypass surgery (6% in the type 1 group), 33% underwent aortic replacement (56% in BAV type 1), and 7% had mitral valve surgery (none with BAV type 1).

There was no significant difference in intraoperative times. The cardiopulmonary bypass time was 112.2 ± 40.3 min in our BAV type 0 group, whereas it was 127.2 ± 48.7 min in the reference BAV type 1 collective. The same applies to aortic clamping time, which was as low as 74.9 ± 28.5 min (BAV type 0) and 70.1 ± 21.4 min (BAV type 1).

With regard to the prosthesis sizes used, there was no significant difference between the two BAV types. The average prosthesis sizes were 25.0 ± 1.9 mm (BAV type 0) and 25.1 ± 1.9 mm (BAV type 1), with 25 mm being the most common size in both groups. In the BAV type 1 group, the 27 mm prosthesis was equivalently frequently used; this was the second most prevalent prosthesis in the BAV type 0 group (Table 3).

### 3.4. Postoperative Data

No intraoperative complications, such as the necessity for the replacement of the prosthetic valve in case of a paravalvular leak, problems with valve deployment, or the need to make use of an assist device, occurred in either group. Immediately after surgery and during hospital stay, no paravalvular leaks or dislocations of the prosthesis occurred in any of the patients. Redo surgery was not performed on any individual.

Further postoperative complications were reported in very few cases in both groups. An important observation is that only four patients in total (three people with BAV type 0 and one person with BAV type 1) required a pacemaker after surgery (*p* = 0.333). Rethoracotomies due to bleeding or tamponade were performed on 13% (two patients) in each group. One patient with BAV type 0 was resuscitated for AV block III and pacer failure and died of multiple organ failure during the further course of hospitalization after recurrent pneumonia, sepsis, and resulting septic shock. (7%). There were no other in-hospital deaths recorded in either cohort. The majority of individuals (60% in the BAV type 0 group; 50% in the BAV type 1 group) were on antibiotic treatment after surgery as infection markers increased, with no individuals developing sepsis or SIRS. Before discharge, the patients were echocardiographed. This showed a low mean maximum gradient of 13.9 ± 5.4 mmHg for BAV type 0 and 17.1 ± 6.7 mmHg for BAV type 1 (Table 4).

### 3.5. Follow-Up

After a three-month follow-up period, mortality for both groups was 0. During this observation period, no further surgical intervention was required, and no one was readmitted to a hospital (Table 4).

In order to put the above results into perspective, the endpoints were compared with a control group consisting of 79 patients who underwent RD SAVR for tricuspid morphology in the same period (Table 5).

## 4. Discussion

The primary challenge regarding bicuspid valves is the asymmetrical configuration and often dilation of the annulus [2,3,4]. These valves are often associated with severe calcification, requiring earlier aortic valve replacement than tricuspid valves [4].

Surgical aortic valve replacement offers the advantage, especially in the case of heavily calcified valves, of allowing for a complete resection of the calcification to be performed. This means that the prostheses can be anchored more firmly than TAVI prostheses. In addition, the implantation of a larger prosthesis is often feasible after decalcification. With conventional aortic valve prostheses, reconstruction of the asymmetric annulus can be easily performed through targeted suturing [1,10,14].

RD valves enable reduced aortic clamping and bypass time with excellent hemodynamic results [15]. Initial findings on the use of sutureless and RD prosthetic valves in BAV morphology have been published in the recent literature, with an increased occurrence of paravalvular leaks reported [10,11,12].

In this single-center study, we compared our experience with RD prostheses in different bicuspid morphologies in terms of overall in-hospital outcomes and immediate and short-term sequelae. In summary, we have reported reliable outcomes, including surgeries with concomitant procedures, with minimal complications regardless of the type of bicuspid valve.

Despite an increased incidence of concomitant procedures in our collectives, our intraoperative times are comparable to those in the existing literature. Intraoperative complications related to valve deployment or conversion to another valve size/prosthesis type, as well as paravalvular leaks or the use of assist devices, did not occur for any subject in either collective. In contrast, Coti et al. reported at least one failed prosthesis positioning in 1.9% of BAV and 2.5% of TAV cases and in 1.6% of TAV cases with an assist device. Miceli et al. also reported one (0.5%) misplaced prosthesis [11,12].

Compared to conventional aortic valve replacement, previous studies have shown an increase in permanent pacemaker implantation (PPI) in RD SAVR [16]. Pacemaker implantation rates range from 6.8% (D’Onofrio et al.) to 12.3% (TRANSFORM study) for a tricuspid morphology [17,18]. Coti et al. reported lower implantation rates for a bicuspid morphology (14.8% TAV vs. 11.4% BAV), while Miceli et al. reported a rate of only 9.7% for bicuspid morphology and RD SAVR [11,12]. Our implantation rates are comparable to those in the existing literature. Overall, we recorded a device rate of 12.9%, with a non-significantly higher number of implants in the BAV type 0 group (20% BAV type 0; 6% BAV type 1).

We also report a total of four (13%) rethoracotomies based on bleeding or tamponade, with no significant difference between the groups (both 13%). However, these values are higher than those reported by Miceli et al. (3.1%) and Coti et al. (1.9% BAV; 3.8% TAV) [11,12].

There were no redo surgeries due to severe prosthesis insufficiency, dislocation, or paravalvular leaks either immediately postoperatively or during our three-month observation period. The same result was reported by Miceli et al., whereas Coti et al. reported moderate- to high-grade paravalvular leaks occurring at rates of 10.8% BAV and 3.9% TAV, and redo surgery based on prosthesis degeneration or endocarditis at rates of 1.2% BAV and 2.4% TAV, respectively, after one year of follow-up [11,12].

The postoperative gradients we measured show that there were no significant differences between the two morphologies, with an average mean gradient (dpmean) of 8.1 ± 3.3 mmHg for BAV type 0 and 8.4 ± 2.9 mmHg for BAV type 1 (*p* = 0.336) and an average maximum gradient (dpmax) of 13.9 ± 5.4 mmHg for BAV type 0 and 17.1 ± 6.7 mmHg for BAV type 1 (*p* = 0.375). These values are slightly lower than those provided by Miceli et al., who reported a dpmean of 10.9 ± 4.4 mmHg and a dpmax of 19.2 ± 7 mmHg. Coti et al. reported comparable values at a 1-year follow-up, with a dpmean 8 mmHg and a dpmax of 19 mmHg (BAV and TAV). This result is also comparable to the values reported by Laufer et al. in tricuspid aortic valves; they reported a dpmean 10.6 ± 4.2 mmHg and a dpmax of 20.0 ± 7.6 mmHg postoperatively before discharge [11,12,15].

The total in-hospital mortality in our case was 0.9%, with only one person in the bicuspid type 0 collective, who died of multi-organ failure during the inpatient stay (3%) (*p* = 0.484). It should be mentioned that this person had a preoperative New Euroscore II of 16.72%. There were no further cases of death in the 3-month follow-up.

These figures are similar to those in the reference literature; Coti et al. reported 0.6% TAV and 0% BAV mortality, while Miceli et al. reported 1.6% all-cause mortality within 30 days. This corresponds to the results of a 5-year follow-up study by Laufer et al. where early postoperative mortality was 1.7% [11,12,15].

It can also be observed that even in comparison with a comparable tricuspid collective, the results are not significantly different. A clear difference was shown between the valve sizes that were used, which were on average larger in the BAV collective. Both mortality and intra- and postoperative complications were similar in both collectives. The rate of pacemaker implantation was not significantly higher in the bicuspid collective. There were no noteworthy findings in the three-month follow-up for either group.

Our data indicate that, so far, RD SAVR is a safe procedure. Comparable postoperative outcomes can be achieved if the BAV morphology is specifically addressed via the exact placement of the leading nadir suture and further precise valve sizing. We did not observe an increased incidence of prosthesis dislocations or paravalvular leaks or a significant difference in pacemaker rates.

As previously reported in other studies, a skilled surgeon experienced in RD implantation is needed to avoid possible pitfalls regarding the approach to different BAV morphologies. For further extension of the indications for bicuspid valves, endocarditis, and redo surgeries with RD valves, profound experience in RD SAVR is required.

## 5. Limitations

The biggest limitation of the study is its small number of participants and use of only single-center data, meaning that the results can only be assessed to a limited extent. Only those patients whose informed consent we received could be included in the study, which meant that patients who could not be contacted had to be excluded from the study. Furthermore, this study was based on retrospective data collection, which was partially incompletely documented. The surgeon was responsible for choosing the type and size of the prosthesis. In the future, AI will be a great help in analyzing patient-specific data and calculating the correct valve prosthesis for this limitation [19].

Another limitation is the currently still limited follow-up time of up to one year, as we have only recently started to routinely perform RD SAVR in bicuspid valves. A long-term follow-up would certainly be interesting to conduct in the future to compare these results with other centers.

## Figures and Tables

**Table 1:** Patient characteristics (*n* = 31).

	Sievers Type 0 Collective (*n* = 15)	Sievers Type 1 Collective (*n* = 16)	*p* (Sievers Type 0 vs. Type 1 Collective)
follow-up, m	15.9 ± 9.1	14.5 ± 10.7	0.593 ^c^
age, y	64.5 ± 7.5	62 ± 8.0	0.700 ^a^
**gender, *n* (%)**			0.013 ^b^
female	2 (13)	9 (56)	
mean New Euroscore II,%	2.9 ± 4	2.1 ± 1.2	0.146 ^a^
arterial hypertension, *n* (%)	13 (87)	8 (50)	0.054 ^d^
diabetes mellitus, *n* (%)	3 (20)	0 (0)	0.101 ^d^
smoking history, active, *n* (%)	2 (13)	2 (13)	1.000 ^d^
alcohol abuse, *n* (%)	1 (7)	0 (0)	0.733 ^d^
obesity (mean BMI), kg/m^2^	26.9 ± 3.8	29.5 ± 7.4	0.059 ^a^
hyperlipoproteinemia, *n* (%)	12 (80)	8 (50)	0.081 ^b^
family history, *n* (%)	4 (27)	2 (13)	0.394 ^d^
peripheral artery disease (pAVK), *n* (%)	1 (7)	1 (6)	1.000 ^d^
cerebral artery disease (cAVK), *n* (%)	2 (13)	1 (6)	0.600 ^d^
chronic lung disease, *n* (%)	0 (0)	1 (6)	1.000 ^d^
pulmonary embolism, *n* (%)	0 (0)	0 (0)	
rheumatic fever, *n* (%)	0 (0)	0 (0)	
congenital heart disease, *n* (%)	0 (0)	0 (0)	
TIA, *n* (%)	0 (0)	0 (0)	
**stroke, *n* (%)**			
embolic	0 (0)	1 (6)	1.000 ^d^
hemorrhagic	0 (0)	0 (0)	
myocardial infarction, *n* (%)	0 (0)	0 (0)	
renal insufficiency dialysis, *n* (%)	1 (7)	0 (0)	0.484 ^d^
low cardiac output syndrome, *n* (%)	0 (0)	0 (0)	
mechanical ventilation, *n* (%)	0 (0)	0 (0)	
cancer, *n* (%)	2 (13)	1 (6)	0.880 ^d^
NYHA > II, *n* (%)	12 (80)	12 (75)	1.000 ^d^
angina pectoris, *n* (%)	4 (27)	2 (13)	0.651 ^d^
syncope, *n* (%)	1 (7)	1 (6)	1.000 ^d^
vertigo, *n* (%)	3 (20)	1 (6)	0.333 ^d^
CHA_2_DS_2_-VASc-Score	2.4 ± 1.5	1.7 ± 1.0	0.200 ^a^
**antiplatelet, *n* (%)**			
ASS	5 (33)	3 (19)	0.433 ^d^
Clopidogrel	3 (20)	0 (0)	0.101 ^d^
ASS + Clopidogrel	1 (7)	0 (0)	0.170 ^d^
anticoagulation, *n* (%)	0 (0)	0 (0)	

Data presented as *n* (%) or mean (±standard deviation). Boldface indicates headlines. ASS—Aspirin, BMI—body mass index, m—months, TIA—transient ischemic attack, and y—year. Prosthesis used was Edwards Intuity Elite. ^a^: *t*-test for independent samples and *t*-test for equality of means <0.05; prior: test for normal distribution (Shapiro–Wilk). ^b^: Chi-square test (Pearson). ^c^: Mann–Whitney test: asymptotic significance. ^d^: Fisher’s exact test (for >20% of values with expected frequency <5).

**Table 2:** Valve-specific data (*n* = 31).

	Sievers Type 0 Collective (*n* = 15)	Sievers Type 1 Collective (*n* = 16)	*p* (Sievers Type 0 vs. Type 1 Collective)
aortic regurgitation, *n* (%)	0 (0)	1 (6)	1.000 ^d^
combined aortic stenosis and regurgitation, *n* (%)	5 (33)	4 (25)	0.433 ^d^
mean left ventricular ejection fraction, %	56.0 ± 10.6	61.7 ± 7.0	0.124 ^a^
mean gradient, mean mmHg	40.8 ± 13.1	47.1 ± 14.5	0.766 ^a^
maximal gradient, mean mmHg	67.0 ± 17.5	79.6 ± 24.3	0.261 ^a^
aortic orifice area, mean cm^2^	0.85 ± 0.29	0.80 ± 0.26	0.781 ^c^
aortic aneurysm, *n* (%)	4 (27)	5 (31)	0.546 ^d^
endocarditis	0 (0)	1 (6)	1.000 ^d^

Data presented as *n* (%) or mean (±standard deviation). Boldface indicates headlines. mmHg—millimeter mercury. Prosthesis used was Edwards Intuity Elite. ^a^: *t*-test for independent samples and *t*-test for equality of means <0.05; prior: test for normal distribution (Shapiro–Wilk). ^c^: Mann–Whitney test: Asymptotic significance. ^d^: Fisher’s exact test (for >20% of values with expected frequency <5).

**Table 3:** Intraoperative data (*n* = 31).

	Sievers Type 0 Collective (*n* = 15)	Sievers Type 1 Collective (*n* = 16)	*p* (Sievers Type 0 vs. Type 1 Collective)
**priority, *n* (%)**			1.000 ^d^
urgent	0 (0)	1 (6)	
partial sternotomy, *n* (%)	1 (7)	3 (19)	0.600 ^d^
**aortic prosthetic size, mean, mm, *n* (%)**	25.0 ± 1.9	25.1 ± 1.9	0.866 ^a^
19	0 (0)	0 (0)	
21	1 (7)	1 (6)	
23	3 (20)	3 (19)	
25	6 (40)	6 (38)	
27	5 (33)	6 (38)	
**concomitant procedures, *n* (%)**			
coronary artery bypass graft	5 (33)	1 (6)	0.083 ^d^
mitral valve	1 (7)	0 (0)	0.484 ^d^
tricuspid valve	0 (0)	0 (0)	
aortic surgery	5 (33)	9 (56)	0.200 ^b^
ventricular septal defect closure	0 (0)	0 (0)	
PFO closure	0 (0)	1 (6)	1.000 ^d^
left atrial appendage closure	1 (7)	0 (0)	0484 ^d^
**intraoperative data, min**			
cardiopulmonary bypass time	112.2 ± 40.3	127.2 ± 48.7	0.520 ^a^
cross-clamp time	74.9 ± 28.5	70.1 ± 21.4	0.547 ^a^

Data presented as *n* (%) or mean (±standard deviation). Boldface indicates headlines. PFO—persistent foramen ovalve. Prosthesis used was Edwards Intuity Elite. ^a^: *t*-test for independent samples, *t*-test for equality of means <0.05; prior: test for normal distribution (Shapiro–Wilk). ^b^: Chi-square test (Pearson). ^d^: Fisher’s exact test (for >20% of values with expected frequency <5).

**Table 4:** Postoperative outcome (*n* = 31).

	Sievers Type 0 Collective (*n* = 15)	Sievers Type 1 Collective (*n* = 16)	*p* (Sievers Type 0 vs. Type 1 Collective)
mean left-ventricular ejection fraction, %	56.4 ± 9.5	61.7 ± 5.8	0.199 ^c^
mean gradient, mean mmHg	8.1 ± 3.3	8.4 ± 2.9	0.336 ^a^
maximal gradient, mean mmHg	13.9 ± 5.4	17.1 ± 6.7	0.375 ^a^
**mortality, *n* (%)**			
in-hospital	1 (7)	0 (0)	0.484 ^d^
during follow-up	0 (0)	0 (0)	
**intraoperative complications, *n* (%)**			
paravalvular leak (aortic)	0 (0)	0 (0)	
valve deployment (aortic)	0 (0)	0 (0)	
extracorporeal membrane oxygenation or extracorporeal life support	0 (0)	0 (0)	
**postoperative complications, *n* (%)**			
reintubation	0 (0)	0 (0)	
CPR, myocardial infarction	1 (7)	0 (0)	0.484 ^d^
renal failure, permanent	1 (7)	0 (0)	0.484 ^d^
major stroke or TIA	0 (0)	0 (0)	
delirium	1 (7)	2 (13)	1.000 ^d^
rethoracotomy (tamponade)	2 (13)	2 (13)	1.000 ^d^
new pacemaker implantation	3 (20)	1 (6)	0.333 ^d^
paravalvular leak	0 (0)	0 (0)	
dislocation of prothesis	0 (0)	0 (0)	
redo surgery	0 (0)	0 (0)	
postoperative infection	9 (60)	8 (50)	0.576 ^b^
endocarditis, sepsis or systemic inflammatory response syndrome	0 (0)	0 (0)	
pneumonia	8 (53)	6 (38)	0.376 ^b^
urogenital	0 (0)	1 (6)	0.504 ^b^
skin	0 (0)	1 (6)	1.000 ^d^
**hospital and intensive care unit stay**			
ventilation time, h	13.3 ± 12.2	12.7 ± 3.9	0.097 ^a^
intensive care unit stay, d	4.6 ± 5.8	3.6 ± 2.2	0.322 ^a^
hospital stay, d	12.1 ± 4.7	14.8 ± 4.3	0.548 ^a^
follow-up complications 3-months, *n* (%)	0 (0)	0 (0)	

Data presented as *n* (%) or mean (±standard deviation). Boldface indicates headlines. CPR—cardiopulmonary resuscitation, d—day, h—hours, mmHg—millimeter mercury, TIA—transient ischaemic attack. Prosthesis used was Edwards Intuity Elite. ^a^: *t*-test for independent samples, *t*-test for equality of means <0.05; prior: test for normal distribution (Shapiro–Wilk). ^b^: Chi-square test (Pearson). ^c^: Mann–Whitney test: asymptotic significance. ^d^: Fisher’s exact test (for >20% of values with expected frequency <5).

**Table 5:** Comparison with TAV collective.

	BAV (*n* = 31)	TAV (*n* = 79)	*p* (BAV vs TAV)
mean New Euroscore II,%	2.40 ± 2.73	2.02 ± 1.69	0.146 ^a^
aortic stenosis, *n* (%)	25 (80.1)	51 (64.6)	0.444 ^b^
aortic regurgitation, *n* (%)	0 (0)	5 (6.3)	0.473 ^b^
combined aortic stenosis and regurgitation, *n* (%)	9 (29)	23 (29.1)	0.709 ^b^
mean preoperative gradient, mean mmHg	44.79 ± 13.67	40.24 ± 16.32	0.086 ^c^
aortic orifice area, mean cm^2^	0.80 ± 0.27	0.84 ± 0.27	0.506 ^a^
**concomitant procedures, *n* (%)**			
coronary artery bypass graft	5 (14.3)	48 (60.8)	<0.001 ^b^
mitral valve	3 (8.6)	8 (10.1)	0.795 ^b^
tricuspid valve	0 (0)	0 (0)	
aortic surgery	13 (37.1)	0 (0)	<0.001 ^b^
aortic prosthetic size, mean, mm, *n* (%)	25.1 ± 2	24.11 ± 1.49	0.003 ^a,c^
cardiopulmonary bypass time	119.7 ± 44.5	111 ± 38.0	0.250 ^a^
cross-clamp time	72.5 ± 24.95	76.9 ± 26.2	0.661 ^a^
mean postoperative gradient, mean mmHg	8.3 ± 3.1	8.85 ± 5.56	0.804 ^c^
**mortality, *n* (%)**			
in-hospital	1 (3.2)	0 (0)	
during follow-up	0 (0)	0 (0)	
valvular complications, *n* (%)	0 (0)	0 (0)	
new pacemaker implantation	3 (8.6)	3 (3.8)	0.362 ^b^
redo surgery	0 (0)	0 (0)	
major stroke	0 (0)	0 (0)	
intensive care unit stay, d	3.8 ± 4.1	3.6 ± 2.5	0.382 ^c^
follow-up complications 3-months, *n* (%)	0 (0)	0 (0)	

Data presented as *n* (%) or mean (±standard deviation). Boldface indicates headlines. Prosthesis used was Edwards Intuity Elite. ^a^: *t*-test for independent samples, *t*-test for equality of means <0.05; prior: test for normal distribution (Shapiro–Wilk). ^b^: Chi-square test (Pearson). ^c^: Mann–Whitney test: asymptotic significance.

## Data Availability

Data are contained within the article. Further data are available on request.
